# The epidemiology of injury and illness amongst athletes at the Indian Ocean Island Games, Mauritius, 2019

**DOI:** 10.17159/2078-516X/2021/v33i1a11211

**Published:** 2021-08-25

**Authors:** D Garnett, A Bholah, B Olivier, J Patricios, Y D’Hotman, K Sunassee, S Cobbing

**Affiliations:** ème Edition des Jeux des Iles de l’Océan Indien, Mauritius; 1 Physiotherapy Department, College of Health Sciences, University of KwaZulu Natal, Durban, South Africa; 2 Department of Physiotherapy, Faculty of Health Sciences, University of Pretoria, Pretoria, South Africa; 3 Comité d’Organisation de la 10; 4 Physiotherapy Department, Faculty of Health Sciences, University of the Witwatersrand, Johannesburg, South Africa; 5 Wits Sport and Health (WiSH), School of Clinical Medicine, Faculty of Health Sciences, University of the Witwatersrand, Johannesburg, South Africa

**Keywords:** surveillance, prevalence, incidences, elite athletes, prevention, multi-sports

## Abstract

**Background:**

The Indian Ocean Island Games is a multi-sport event that occurs every four years and includes athletes from seven islands of the Indian Ocean, namely, Comoros, Reunion, Mayotte, Madagascar, Maldives, Seychelles, and Mauritius.

**Objectives:**

This study aims to describe the injury and illness epidemiology of the athletes participating during the 2019 Indian Ocean Islands Games.

**Methods:**

This prospective cohort study recorded injury and illness cases from athletes who competed in these Games. All medical physicians received detailed instructions and training on data collection using an injury report form. All athletes (minors and adults) who provided consent, or consent given from the minors’ guardians, were included in this study. Athletes who did not provide consent for this study were excluded.

**Results:**

Athletes (n = 1 521; 531 women and 990 men) reported 160 injuries (injury incidence rate of 11%) and 85 illnesses (illness incidence rate of 6%). The percentage of distribution of injuries were highest in football and basketball. Most injuries occurred during competition, compared with training, joint sprains were the most common type of injury (28%), followed by muscle strains (19%). Men suffered most of the injuries (79% vs. 21% for women). Similarly, men sustained more illnesses than women (57% vs. 43%). Most illnesses affected the respiratory system (67%), and infection was the most common cause of illness (84%) in participating athletes.

**Conclusion:**

These findings are similar to previous events in other parts of the world. However, unique ailments, not previously reported on, were discovered. Epidemiological data from this study can be inferred to athletes who compete in similar multi-sport events in the Indian Ocean region.

The 10^th^ Indian Ocean Island Games (IOIG) were held in Mauritius from 19–28 July 2019. This multi-sport event occurs every four years between participating athletes from seven of the Indian Ocean islands, namely, Comoros, Reunion, Mayotte, Madagascar, Maldives, Seychelles, and Mauritius. The 14 sporting codes represented included football, basketball, rugby sevens, judo, table tennis, volleyball, beach volleyball, swimming, yachting, weightlifting, boxing, athletics, cycling and badminton. In almost half a century of competition, no previous injury or illness research has been conducted for this event, and little is known of how injuries in these athletes compare to athletes in other parts of the world. Surveillance studies towards improving athlete welfare are supported by the International Olympic Committee (IOC), and they allow a snapshot of current injuries and illness, which are essential in the preparation of future events.^[1]^

Injury surveillance during similar sporting events have been documented using standardised injury reporting since the 2004 Olympic Games in Athens.^[2]^ The incidence of injury at both the Summer and Winter Olympic Games appears to be increasing,^[2–8]^ apart from the Rio Games, where compliance in reporting by medical staff was unaccountably lower than recent studies (74% in Rio, 99.7% in Sochi and 96% in London).^[5–6]^ Interestingly, the incidence of athletic injury and illness varied substantially between the different sporting codes over the same period. This finding calls for bespoke prevention strategies tailored to the differing demands placed on the athletes of the specific sports.^[2–5]^ This planning should also include strategies to support athletes when exposed to different climates. Specific to this study are the warm and humid conditions of the tropical island of Mauritius.

This research is novel as it is the first such project that captures the injury and illness data from all athletes participating at the Indian Ocean Island Games – a first in the Games’ 40-year history and a first for sports in this tropical region – as well as comparing these data to other international research. Continued surveillance of injury and illness at subsequent Indian Ocean Island Games will allow the establishment of customised and relevant prevention strategies to enhance athlete welfare in this region.

## Methods

### Study design and setting

The study design was a prospective surveillance study of injuries and illnesses occurring during the Indian Ocean Island Games (IOIG) 2019 on the Mauritius Island. The definitions and methodologies are described in detail in this manuscript and have been adopted from similar previous research to allow for the comparison of results as per recommendations by these authors.^[2,5,9]^

### Ethical considerations

Permission to conduct the research study was granted by the Games local organising committee, namely the Comite d’Organisation de la 10eme Edition des Jeux des Iles de L’Ocean Indien (COJI 2019). Ethical clearance was obtained from Mauritian and South African research ethics committees (BREC: RECIP471/19/ WITS: M190444). Informed consent was obtained from the respective team authorities and all individual participants included in the study. Athletes who were considered minors gave informed assent and informed consent were obtained from their respective guardians. Athletes who did not wish to participate or did not provide informed consent were excluded. All forms were translated and presented in French and English.

### Participants

All athletes were provided with study information packs on registration and invited to participate in the research study. All data were coded to protect confidentiality and ensure privacy. All athletes had access to identical treatment, whether part of the research study or not.

### Injury report form

The research instrument was the injury report form (Appendix A), which was a simple single page form with references and further information on the back of the form. Sections of the injury reporting form included:

- Accreditation number - which detailed anthropometric and demographic data of athletes.
- Injury - injury type, cause of injury, date, time and site of injury, injured body part and cause.
- Illness - diagnosis, affected system, main symptom(s), probable cause.
- Severity of injury or illness - time out of competition or training calculated in days.
- Sports and event type - as described by the technical department of Comité d’Organisation de la 10^ème^ Edition des Jeux des Iles de l’Océan Indien (COJI) in 2019.

### Definitions

An injury was defined as any new musculoskeletal complaint that occurred during competition or training within the period of the tournament and that received medical attention, regardless of the consequences concerning absence from competition or training. This injury definition includes five aspects: (i) all injuries that received medical attention, (ii) newly incurred injuries, (iii) injuries occurring during competition or training, (iv) injuries occurring during the period of the tournament, and (v) exclusion of other illnesses and diseases.^[1,4,9]^ All injuries and illnesses were coded to ensure a standard injury or illness definition. An illness was defined as any physical symptom that required medical attention or prevented an athlete from taking part in training and competition.^[10]^

### Procedures

The sports federations and medical representatives of all participating countries were requested to participate and were electronically provided with information regarding the study one month before the IOIG. All host nation medical staff received training on the completion of the injury report form in training ahead of the IOIG. On the eve of the IOIG, all visiting physicians responsible for athletes received a printed booklet with information about the study and attended a pre-study workshop. During this meeting, the aims of the study were presented, practical components discussed, the roles and responsibilities defined, and the contact details of the research team were provided. Detailed instructions on how to complete the injury reporting form were given, with examples, on how to report an athlete’s injury and/or illness.^[1,7,9]^

Thus, all injuries and illnesses at the IOIG were diagnosed and reported by qualified and trained medical personnel to ensure valid information was provided regarding the characteristics of the injury and to allow a comparable standard of data.

The researchers conducted injury surveillance over 11 days (19–28 July 2019). During the tournament, members of the research team visited the medical physicians of the respective countries at the team hotels to ensure compliance and discuss any problems that they may have encountered. The sequence of daily data collection procedures included the following six steps; **Step 1**: a single injury reporting form was completed for every injury that was presented to each doctor (team and local). Thus, a single participant could have had multiple forms for polytrauma events. **Step 2**: the completed forms were collected by the principal researcher and scanned for missing information. **Step 3:** if possible, errors discussed with the reporting physician, and if there were no errors, double entries were identified. **Step 4:** if there were double entries, then the team physician’s form would be used. **Step 5:** data were captured onto a password encrypted electronic form on a password accessed computer. **Step 6:** completed hard copy forms were placed into a password-protected safe to ensure the anonymity of participants.

### Statistical analysis

In cases where an injury or illness was duplicated in reporting by the IOIG medical teams and team physicians, the most complete data source was retained.^[3]^ The illness and injury incidences (i) were calculated according to the formula *i = n/e*, where *n* is the number of injuries or illnesses in competition or training and *e* is the total number of participating athletes during the total study period of 11 days. Descriptive data was captured from the hard copies to electronic format for coding using Microsoft Excel software. Statistical analysis was performed using GraphPad Prism 9.1.2 software and checked by a statistician from University of KwaZulu-Natal.

## Results

### Participants

In total, 1 521 athletes were registered for the competition; this included 531 women and 990 men. The ages of the athletes ranged from 10–59 years (mean: 25.2 years). The average age of female athletes was 23.8 years (range 10.2 – 59.5 years) and 25.9 years for male athletes (12.6 – 57.2).

### Incidence of injury

The 1 521 athletes sustained a total of 160 injuries, resulting in an injury incidence rate of 10.5% (10.5 injuries per 100 athletes) (Table 1). Most injuries occurred during competition when compared with training (57% vs. 43%). Most of the injuries were suffered by male athletes (79% vs. 21%).

### Injury location and type

The percentage of the body region most frequently injured was the lower limb (63%), followed by the upper limb (21%), and head/torso (16%). The most common sites of injury were the thigh (20%), followed by the knee (18%), calf (11%), shoulder (9%), and ankle (8%) (Table 2).

The most common types of injury reported were joint sprains (28%), muscle strains (19%), muscle cramps or spasms (16%), contusions/haematomas (13%), lacerations (9%), tendinosis/tendinopathies (5%), concussions (3%), fractures (3%), other bone injuries (2%), ligamentous ruptures (1%), arthritis/synovitis/ bursitis (1%), dental injuries (1%) and other injuries (1%).

### Injury mechanisms and circumstances

The mechanism of a sustained injury was most commonly due to contact with other athletes (collisions) (28%), overuse with sudden onset of injury (19%), non-contact trauma (17%), overuse with gradual onset (17%), contact with a stagnant object (8%), contact with a moving object (5%), recurrence of previous injuries (3%), violation of rules (3%), field of play conditions (1%), and a player being tackled (1%). Tacklers, being the defending players without possession of the ball, had no reported injuries (0%).

### Illness distribution and the affected system

The 1 521 athletes reported a total of 85 illnesses, resulting in an illness incidence of 6% (6 illnesses per 100 athletes). The respiratory system was the most affected system as shown in Table 3. Overall, men reported more illnesses than women (57% vs. 43%). The most frequently reported cause of illness in athletes was infection (84%), followed by environmental causes (5%), pre-existing conditions (5%), exercise-induced (4%), others (2%), and reaction to medication (1%).

## Discussion

The primary finding of this research is that the injury incidence was 10.5 injuries sustained per 100 athletes, and 6 illnesses sustained per 100 athletes. As this was the initial study, no previous IOIG data were available for direct comparison. Thus, injury data from previous surveillance research by the International Olympic Committee (IOC) were used to allow comparison of similar athletes at multi-event tournaments over a fixed study period using similar methods. Furthermore, IOIG illness data were compared to the Olympic Games’ surveillance studies since 2010, as this was the first official IOC commencement of methodology to include illness surveillance.

### Incidences of injury

**Fig. 1:**
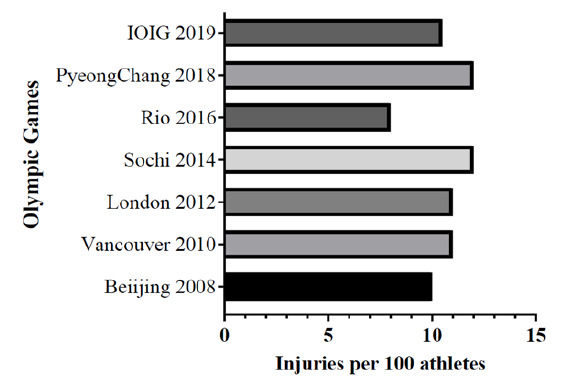
Incidence of injury at previous Olympic Games^[3–8]^ and the Indian Ocean Island Games (IOIG) 2019

The overall incidence of injury to athletes at IOIG 2019 is comparable to previously reported injuries at Beijing, Vancouver, London, and Rio Games and a lower injury incidence at the Sochi and PyeongChang Games (Fig.1).^[3–8]^ The weather in Mauritius is similar to that experienced in the Summer Olympic Games, when data is compared from similar sporting codes. The incidence of injury is similar at the IOIG 2019 (10.5 injuries sustained per 100 athletes vs. 8–11 injuries sustained per 100 athletes) when compared with the Winter Olympic Games (10–12 injuries sustained per 100 athletes).^[4,6,8]^ However, male athletes suffered more injuries than female athletes at the IOIG 2019, in contrast to previous findings from the Winter Olympic Games in Vancouver 2010, Sochi 2014 and PyeongChang 2018.^[4,6,8]^ The vastly different nature of the sporting codes at the IOIG and Summer Olympic Games versus the Winter Olympic Games may account for some of these differences, ^[3–8]^ but serial research at future IOIGs is required in order to identify accurate trends.

### Injury location and circumstances

The distribution of injuries during competition and training at IOIG was similar to previous studies in the Summer Olympic Games (Table 4).^[5, 7]^ However; they are contrast to the Winter Olympic Games, where training injuries were higher than competition injuries (Table 4). ^[4,6,8]^ The most common sites of injury at the IOIG 2019 were similar to previous studies for Vancouver 2010 (head 19–20%; knee 10–16%), Rio 2016 (knee 12%; thigh 10%) and PyeongChang 2018 (knee 14%; ankle 9%).^[4,7,8]^ A clear indication shows the knee being the most injured site during all Olympic Games tournaments over the past decade, possibly due to large rotational demands placed on a primarily hinge-type joint.^[10]^ This valuable information allows for focused knee injury prevention initiatives for participating athletes.

### Type and cause of injuries

At the IOIG 2019, the three most common causes of injury were similar with previous results from Beijing 2008, Vancouver 2010, London 2012, Sochi 2014, and Rio 2016 (Table 4).^[3–7]^ In PyeongChang 2018, the most common cause of injury was contact with a stagnant object and non-contact trauma. This is similar to previous Winter Olympic Games, which may be an indication of the local conditions of the host venues and specific equipment required for these Games.^[4,6,8]^ For example, these conditions may add an element of risk not seen in other venues. Vancouver had many injuries with stagnant objects in the bobsleigh run and skiing/snowboarding slopes, and Sochi had many injuries caused by the snowboard halfpipe.^[4]^ The nature of these sports necessitate a low friction coefficient of ‘playing’ surfaces and conveys an increased risk of injury. Interestingly, at IOIG 2019 in Mauritius in a tropical climate, ‘marine coral’ was identified as “field of play conditions’ which resulted in lacerations of the lower leg and feet of some of the athletes. Statistical analysis of these injury data was performed using Pearson’s correlation (*P*=0.05, 95% CI). The values were significant for most of the Olympic Games except for Sochi 2014 (Beijing *P*=0.0162; Vancouver *P*=0.0464; London *P* =0.0053; Sochi *P*=0.2096; Rio *P*=0.0003; PyeongChang *P*=0.0349). Caution should be exercised when interpreting these results as different methodologies between studies resulted in the item “contact with another athlete” not being available as an option for injury mechanism/cause in PyeongChang 2018.^[8]^

Knowledge and awareness of concussion have improved significantly over the past decade,^[11–13]^ and effective preventative strategies such as protective equipment may account for the lower number of reported concussions during IOIG 2019. This incidence falls within the mid-range (range = 0.06% – 0.8%) of previously reported research in Beijing, Vancouver, London, Sochi, Rio and PyeongChang. ^[3–8]^

### Incidence and distribution of illnesses

The overall incidence of illness to athletes at IOIG 2019 is lower than previously reported at studies at the Olympic Games as shown in Table 5.^[4–6,8]^ However, in contrast to previous Olympic Games, men reported more illness than women at IOIG.^[5–8]^ Although it is important to identify that more male athletes were included in the study, than female athletes. Most athlete illnesses affected the respiratory and gastrointestinal systems, corresponding to previous Winter and Summer Olympic Games (range 47–70% respiratory system; 13–21% gastrointestinal system).^[4–8]^ Interestingly, a trend is forming with higher infection rates and respiratory illness at the Winter Olympic Games. The winter conditions exposed participating athletes to vastly different external factors than the weather conditions in Mauritius in 2019. The risk of upper respiratory tract illness may be increased from repeated cold air exposure and supports the higher incidence resulting from exercise-induced or environmental factors in the Winter Olympic Games, compared to IOIG.^[14,15]^ In contrast, infection was the most common cause of illness at IOIG 2019. This was significantly higher than previously reported studies,^[4–8]^ and may be representative of the humid tropical environment that the athletes were exposed to. Statistical analysis of illness data was performed using Pearson’s correlation (*P*=0.05, 95% CI). The values were significant for all Olympic Games, except for London 2010 (Beijing no illness data collected; Vancouver *P*=0.0136; London *P*=0.0849; Sochi *P*=0.0394; Rio *P*=0.0319; PyeongChang *P*=0.0183).

When comparing the sports at the Summer Olympic Games, the incidence of illness varied substantially between sporting codes. Athletes participating in track-and-field events reported significantly more illnesses than in previous Summer Olympic Games in London (7%) and Rio (6%) respectively.^[5,7]^ By contrast, illnesses for footballers was lower at the IOIG 2019 than during the London and Rio Games (8% vs. 12% and 12% respectively).^[5,7]^

### Limitations

There are limitations to the methodology of this study that are common to previous similar research. Attending medical staff and athletes may not have engaged and participated in the research as intended. Measures to improve compliance by explaining the benefits of the research were utilised. However, many athletes were not accustomed to medical research procedures, and some athletes chose not to participate in the study. Although research study information was supplied both electronically and in printed form to the international sporting federations, the authors are unsure whether this information was disseminated to all athletes. Furthermore, the compliance of medical staff to complete the injury reporting forms effectively could not be guaranteed as it placed an additional administrative burden on their clinical duties. Previous injuries or illnesses were not accounted for and could be a factor to consider in future studies. Lastly, small changes in reporting methodology by different host nations and local organising committees in previous studies may result in data that is not entirely comparable.

### Recommendations

This research presents the first account of mechanisms and contexts of injuries and the incidences and distribution of illnesses in the 40-year history of the IOIG. The information on the mechanisms and contexts for the injuries and illnesses allows for the evaluation of the 2019 medical services and guides of future organising committees of the IOIG to ensure the most beneficial allocation of resources. This unique setting exposes athletes to conditions not seen in other parts of the world where some conditions may be beneficial or harmful to the athletes. The continued collection of data at these Games will allow more precise preparation for multi-event sporting competitions that will be unique to the region. To ensure greater research clarity, athlete understanding, and improved athlete participation, future studies of this nature in this region should explore alternative methods of information sharing that include electronic/social media/app or peer-based methods. Furthermore, future studies should allow sufficient budgeting and allocation of resources to enable medical administrators to act as dedicated data capturers in order to ensure a higher standard of compliance and data collection.

## Conclusion

An injury and illness surveillance system for multiple sports events was adapted from recent Olympic Games, and developed and implemented for the 10^th^ Indian Ocean Island Games held in Mauritius in 2019. This was the first study to report on the epidemiology of injuries and illnesses at these Games and provides important information for the planning of subsequent multi-national multi-sport events in this region. Just over ten percent (10.5%) of all athletes sustained an injury and 6% sustained an illness during the Games, similar to the information from recent Olympic Games. Continued analyses of illness risk factors and injury mechanisms are essential to direct future athlete welfare policies. Early recommendations encourage further research in developing injury prevention programmes to safeguard the needs of the athlete and prevent illnesses by reducing infections to the upper respiratory tract. This study is the first step in developing informed injury and illness prevention initiatives to allow athletes to perform at their highest level while minimising their health risks, and comes at a time of heightened awareness and need to reduce respiratory illness.

## Key points

- Emerging data from this established traditional tournament reveal novel insights into the athletes of the Indian Ocean.
- A trend is forming showing how elite athletes display similar rates of illnesses and injuries in multi-sport events in different regions of the world.
- Future studies are recommended to specifically assess the unique risk factors to individual regions to better prepare athletes and the medical teams who support these athletes.

## Supplementary Information



## Figures and Tables

**Table 1:** Distribution of injuries and illnesses in all athletes per sport at the Indian Ocean Island Games (IOIG) 2019

Sport	Injuries			Illnesses		
	All athletes (n = 160)	Male (n = 127)	Female (n = 33)	All athletes (n = 85)	Male (n = 49)	Female (n = 36)
**Football**	38 (24)	38 (24)	0 (0)	7 (8)	6 (7)	1 (1)
**Basketball**	23 (14)	13 (8)	10 (6)	5 (6)	2 (2)	3 (4)
**Athletics**	18 (11)	14 (9)	4 (3)	18 (21)	9 (11)	9 (11)
**Volleyball**	18 (11)	13 (8)	5 (3)	13 (15)	7 (8)	6 (7)
**Judo**	13 (8)	7 (4)	6 (4)	1 (1)	0 (0)	1 (1)
**Rugby 7’s**	13 (8)	13 (8)	0 (0)	6 (7)	6 (7)	0 (0)
**Sailing**	9 (6)	8 (5)	1 (<1)	3 (4)	2 (3)	1 (1)
**Badminton**	8 (5)	5 (3)	3 (2)	6 (7)	3(4)	3 (4)
**Boxing**	8 (5)	8 (5)	0 (0)	7 (8)	6 (7)	1 (1)
**Weightlifting**	4 (3)	2 (1)	2 (1)	6 (7)	3 (4)	3 (4)
**Table Tennis**	2 (1)	1 (<1)	1 (<1)	6 (7)	2 (3)	4 (5)
**Cycling**	2 (1)	2 (1)	0 (0)	1 (1)	1 (1)	0 (0)
**Swimming**	2 (1)	2 (1)	0 (0)	6 (7)	2 (3)	4 (5)
**Beach Volleyball**	2 (1)	1 (<1)	1 (<1)	0 (0)	0 (0)	0 (0)

Data are expressed as n (%).

**Table 2:** Injury location of athletes at the Indian Ocean Island Games (IOIG) 2019

Body region or part	All athletes (n = 160)	Males (n = 126)	Females (n = 34)
**Lower limb**	100 (63)	81 (51)	19 (12)
**Upper limb**	34 (21)	24 (15)	10 (6)
**Head/torso**	26 (16)	21 (13)	5 (3)

**Thigh**	32 (20)	29 (18)	3 (2)
**Knee**	29 (18)	19 (12)	10 (6)
**Calf**	18 (11)	16 (10)	2 (1)
**Shoulder**	15 (9)	8 (5)	7 (4)
**Ankle**	13 (8)	9 (6)	4 (3)
**Lower back**	8 (5)	6 (4)	2 (1)
**Face**	7 (4)	6 (4)	1 (<1)
**Hand**	6 (4)	5 (3)	1 (<1)
**Thumb**	5 (3)	5 (3)	0 (0)
**Neck**	4 (3)	4 (3)	0 (0)
**Finger**	4 (3)	4 (3)	0 (0)
**Head**	4 (3)	3 (2)	1 (<1)
**Foot**	4 (3)	3 (2)	1 (<1)
**Elbow**	3 (2)	1 (<1)	2 (1)
**Upper arm**	2 (1)	2 (1)	0 (0)
**Achilles tendon**	1 (<1)	1 (<1)	0 (0)
**Groin**	1 (<1)	1 (<1)	0 (0)
**Hip**	1 (<1)	1 (<1)	0 (0)
**Pelvis**	1 (<1)	1 (<1)	0 (0)
**Thoracic region**	1 (<1)	1 (<1)	0 (0)
**Abdomen**	1 (<1)	1 (<1)	0 (0)

Data are expressed as n (%).

**Table 3:** Distribution of athlete illness affected systems at the Indian Ocean Island Games (IOIG) 2019

Affected system	All athletes (n = 85)	Males (n = 49)	Females (n = 36)
**Respiratory**	57 (67)	36 (42)	21 (25)
**Gastrointestinal**	15 (18)	11 (13)	4 (5)
**Dermatologic**	5 (6)	0 (0)	5 (6)
**Cardiovascular**	4 (5)	1 (1)	3 (4)
**Allergic**	1 (1)	0 (0)	1 (1)
**Metabolic**	1 (1)	0 (0)	1 (1)
**Neurologic**	1 (1)	0 (0)	1 (1)
**Dental**	1 (1)	1(1)	0 (0)

Data are expressed as n (%).

**Table 4:** Injury characteristics for participating athletes

Cause of injury	Beijing 2008	Vancouver 2010	London 2012	Sochi 2014	Rio 2016	PyeongChang 2018	IOIG 2019
**Contact with another athlete**	33	15	14	-	28	-	28
**Non-contact trauma**	20	23	20	13	21	18	17
**Overuse with sudden onset**	13	-	25	-	19	-	19
**Contact with a stagnant object**	-	22	-	25	-	31	-
**Concussion**	0.11	0.8	0.06	0.4	0.11	0.17	0.26
**Injured during competition**	73	46	55	35	59	46	57
**Injured during training**	25	54	45	63	37	48	43

Data are expressed as % of participating athletes at each games^[3–8]^. IOIG, Indian Ocean Island Games; -, data not available.

**Table 5:** Incidence and distribution of illnesses

	Beijing 2008	Vancouver 2010	London 2012	Sochi 2014	Rio 2016	PyeongChang 2018	IOIG 2019
**Illness incidence**	-	7	7	8	5	9	6
**Respiratory illness**	-	62	41	64	47	70	67
**Gastrointestinal illness**	-	20	16	11	21	13	18
**Illness for Females**	-	-	60	64	59	60	44
**Illness for Males**	-	-	40	36	41	40	56
**Infection**	-	64	46	58	56	63	84

Data are expressed as % of participating athletes at each games^[3–8]^. IOIG, Indian Ocean Island Games; -, data not available. Illness incidence (i) is calculated as i = n/e, where n is the number of illnesses in competition or training and e is the total number of participating athletes during the total study period of 11 days
