# Supplementary material for: The epidemiology of injury and illness amongst athletes at the Indian Ocean Island Games, Mauritius, 2019
Source: S Afr J Sports Med. 2021 Aug 25;33(1):v33i1a11211. doi: 10.17159/2078-516X/2021/v33i1a11211 (PMC9924607; doi:10.17159/2078-516X/2021/v33i1a11211)
Supplement: Supplementary file 1 [file 2078-516X-33-v33i1a11211-s001.pdf]

The epidemiology of injury and illness amongst athletes at the Indian Ocean Island Games, Mauritius, 2019

Appendix A

Daily Report on Injury & Illnesses

10e JIOI MAURICE 2019

Report by (name)________________________________ Date____________________________

Please report (1) All injuries and (2) all illness newly incurred in competition or during training regardless of the consequences with respect to absence from competition or training. The information provided is for medical research purposes and will be treated confidentially.

☐ Mauritius ☐ Seychelles ☐ Comoros ☐ Madagascar ☐ Mayotte ☐ Maldives ☐ Réunion ☐ Sri Lanka

See reverse for definitions and codes

Injury

☐ No injury in any athlete of our team today

Athlete's accreditation no.<br>12345678910		Sport and event<br>Athletics, 100m (women)		Round/heat or training<br>Quarter final/1st heat		Date & time of injury<br>24/07/19 14h30
Injured body part<br>Wrist, left	Code<br>15	Type of injury<br>Sprain	Code<br>8	Cause of injury<br>Slipped and fell	Code<br>3	Absence in days<br>10

Athlete's accreditation no.		Sport and event		Round/heat or training		Date & time of injury
Injured body part	Code	Type of injury	Code	Cause of injury	Code	Absence in days

Athlete's accreditation no.		Sport and event		Round/heat or training		Date & time of injury
Injured body part	Code	Type of injury	Code	Cause of injury	Code	Absence in days

Athlete's accreditation no.		Sport and event		Round/heat or training		Date & time of injury
Injured body part	Code	Type of injury	Code	Cause of injury	Code	Absence in days

Athlete's accreditation no.		Sport and event		Round/heat or training		Date & time of injury
Injured body part	Code	Type of injury	Code	Cause of injury	Code	Absence in days

Illness

☐ No illness in any athlete of our team today

Athlete's accreditation no.<br>12345678911		Sport and event<br>Football (men)		Diagnosis<br>Tonsillitis/cold		Occurred on (date)<br>24/07/19
Affected system<br>Respiratory	Code<br>1	Main symptoms<br>Fever, pain	Code<br>1,2	Cause of illness<br>infection	Code<br>2	Absence in days<br>2

Athlete's accreditation no.		Sport and event		Diagnosis		Date & time of injury
Affected system	Code	Main symptoms	Code	Cause of illness	Code	Absence in days

Athlete's accreditation no.		Sport and event		Diagnosis		Date & time of injury
Affected system	Code	Main symptoms	Code	Cause of illness	Code	Absence in days

Please use additional forms if necessary.

10e JIOI MAURICE 2019

Codes and classifications

Estimated duration of absence from training or competition (in days)

Please provide an estimate of the number of days that the athlete will not be able to undertake his/her normal training programme or will not be able to compete.

0 = 0 days<br>1 = 1 days<br>2 = 2 days	7 = 1 week<br>14 = 2 weeks<br>21 = 3 weeks	28 = 4 weeks<br>>30 = more than 4 weeks<br>>180 = 6 months or more

Sport and event: Please sate the event of injury – For example: swimming – 4 x 100m freestyle relay(women)

For Injuries

Round, heat or training

If the injury occurred during competition, please state the round (e.g. heats, qualification B, final)
If the injury occurred on another occasion, please specify whether it was training, warm up or other.

Injured body part (location of injury)

Head and trunk	Upper extremity	Lower extremity
1 face<br>2 head<br>3 neck / cervical spine<br>4 thoracic spine / upper back<br>5 sternum / ribs<br>6 lumbar spine / lower back<br>7 abdomen<br>8 pelvis / sacrum / buttock	11 shoulder / clavicle<br>12 upper arm<br>13 elbow<br>14 forearm<br>15 wrist<br>16 hand<br>17 finger<br>18 thumb	21 hip<br>22 groin<br>23 thigh (a: anterior / p: posterior)<br>24 knee (m: medial / l: lateral)<br>25 lower leg (a: anterior / p: posterior)<br>26 Achilles tendon<br>27 ankle (m: medial / l: lateral)<br>28 foot/toe

Type of Injury (diagnosis)

1 Concussion (regardless of loss of consciousness<br>2 fracture (traumatic)<br>3 stress fracture (overuse)<br>4 other bone injuries<br>5 dislocation, subluxation<br>6 Tendon rupture<br>7 Ligamentous rupture	8 sprain<br>(injury of joint and or/ligaments)<br>9 lesion of meniscus or cartilage<br>10 strain / muscle rupture / tear<br>11 contusion / haematoma / bruise<br>12 tendinosis/ tendinopathy<br>13 arthritis / synovitis / bursitis<br>14 fasciitis / aponeurosis injury	15 impingement<br>16 laceration / abrasion /skin lesion<br>17 dental injury / broken tooth<br>18 nerve injury / spinal cord injury<br>19 muscle cramps or spasm<br>20 other – please describe

Cause of Injury

1 overuse (gradual onset)<br>2 overuse (sudden onset)<br>3 non-contact trauma<br>4 recurrence of previous injury<br>5 contact with another athlete	6 Contact: moving object<br>7 Contact: stagnant object<br>8 Violation of rules (obstruction, pushing)<br>9 field of play conditions	10 weather conditions<br>11 equipment failure<br>12 tackler<br>13 one being tackled<br>14 other – please describe

For Illnesses

Affected system

1 respiratory / ear, nose, throat<br>2 gastro-intestinal<br>3 uro-genital / gynaecological<br>4 cardio-vascular	5 allergic / immunological<br>6 metabolic / endocrinological<br>7 haematological<br>8 neurological / psychiatric	9 dermatologic<br>10 musculo-skeletal<br>11 dental<br>12 other – please describe

Main symptom(s)

1 fever<br>2 pain<br>3 diarrhoea, vomiting<br>4 dyspnoea, cough	5 palpitations<br>6 hyper-thermia<br>7 hypo-thermia<br>8 dehydration	9 syncope, collapse<br>10 anaphylaxis<br>11 lethargy, dizziness<br>12 other – please describe

Cause of illness / symptom(s)

1 pre-existing (e.g. asthma, allergy)<br>2 infection	3 exercise-induced<br>4 environmental	5 reaction to medication<br>6 other – please describe
